# Effect of Canagliflozin on Renal Threshold for Glucose, Glycemia, and Body Weight in Normal and Diabetic Animal Models

**DOI:** 10.1371/journal.pone.0030555

**Published:** 2012-02-15

**Authors:** Yin Liang, Kenji Arakawa, Kiichiro Ueta, Yasuaki Matsushita, Chiaki Kuriyama, Tonya Martin, Fuyong Du, Yi Liu, June Xu, Bruce Conway, Jamie Conway, David Polidori, Kirk Ways, Keith Demarest

**Affiliations:** 1 Johnson & Johnson Pharmaceutical Research & Development, LLC, Spring House, Pennsylvania, United States of America; 2 Pharmacology Laboratory, Mitsubishi Tanabe Pharma Corporation, Toda, Saitama, Japan; 3 Johnson & Johnson Pharmaceutical Research & Development, LLC, La Jolla, California, United States of America; 4 Johnson & Johnson Pharmaceutical Research & Development, LLC, Raritan, New Jersey, United States of America; University of Padova Medical School, Italy

## Abstract

**Background:**

Canagliflozin is a sodium glucose co-transporter (SGLT) 2 inhibitor in clinical development for the treatment of type 2 diabetes mellitus (T2DM).

**Methods:**

^14^C-alpha-methylglucoside uptake in Chinese hamster ovary-K cells expressing human, rat, or mouse SGLT2 or SGLT1; ^3^H-2-deoxy-d-glucose uptake in L6 myoblasts; and 2-electrode voltage clamp recording of oocytes expressing human SGLT3 were analyzed. Graded glucose infusions were performed to determine rate of urinary glucose excretion (UGE) at different blood glucose (BG) concentrations and the renal threshold for glucose excretion (RT_G_) in vehicle or canagliflozin-treated Zucker diabetic fatty (ZDF) rats. This study aimed to characterize the pharmacodynamic effects of canagliflozin in vitro and in preclinical models of T2DM and obesity.

**Results:**

Treatment with canagliflozin 1 mg/kg lowered RT_G_ from 415±12 mg/dl to 94±10 mg/dl in ZDF rats while maintaining a threshold relationship between BG and UGE with virtually no UGE observed when BG was below RT_G_. Canagliflozin dose-dependently decreased BG concentrations in db/db mice treated acutely. In ZDF rats treated for 4 weeks, canagliflozin decreased glycated hemoglobin (HbA1c) and improved measures of insulin secretion. In obese animal models, canagliflozin increased UGE and decreased BG, body weight gain, epididymal fat, liver weight, and the respiratory exchange ratio.

**Conclusions:**

Canagliflozin lowered RT_G_ and increased UGE, improved glycemic control and beta-cell function in rodent models of T2DM, and reduced body weight gain in rodent models of obesity.

## Introduction

Due in part to the increasing prevalence of obesity and the aging of the global population, type 2 diabetes (T2DM) is becoming an increasingly prevalent disorder [1]. While lifestyle interventions are effective means to improve glycemic control, as beta-cell loss ensues and illness progresses, patients require increasingly complex therapies involving combinations of antihyperglycemic agents, including insulin, in order to attain optimal glycemic control. Currently approved antihyperglycemic agents act by increasing insulin secretion, enhancing insulin sensitivity, or reducing glucose absorption. Despite the availability of a pharmacologic armamentarium containing a number of efficacious antihyperglycemic agents, fewer than 50% of patients achieve glycemic treatment goals set forth by expert societies [2].

In a normoglycemic person, approximately 180 grams of blood glucose (BG) is filtered by the glomerulus and is resorbed in the proximal tubule such that urinary glucose excretion (UGE) is negligible [3], [4]. As plasma glucose concentrations increase above normal, UGE remains negligible until the filtered glucose load begins to saturate the capacity of the renal glucose transporters. The plasma glucose concentration at which this occurs is called the renal threshold for glucose excretion (RT_G_). The majority of renal glucose resorption is mediated by sodium glucose co-transporter 2 (SGLT2), a high-capacity, low-affinity glucose transporter localized in the luminal membrane of early proximal renal tubular cells [3], [4]. Once transported by SGLT2 into the tubular cell, glucose is transported down its concentration gradient and into the renal interstitium by the facilitative glucose transporter 2 (GLUT2) [3], [4]. The filtered glucose not resorbed by SGLT2 is subsequently resorbed in more distal portions of the proximal tubule by SGLT1, a high-affinity, low-capacity glucose transporter and is transported from the proximal tubular cell into the renal interstitium by the facilitative GLUT1 [3], [4]. Although SGLT2 and SGLT1 are believed to transport the vast majority of glucose from the tubular lumen, other less well-characterized glucose transporters may also be involved in renal glucose resorption [5].

SGLT2 is expressed almost exclusively in the proximal tubule of the kidney [3], [4]. Mutations in the *SLC5A2* gene encoding SGLT2 are found in persons with familial renal glucosuria (FRG) [6]; FRG is considered a benign condition, with affected individuals exhibiting glucosuria in the absence of hyperglycemia without alteration in other proximal tubular functions [6]. Recently, an SGLT2-null mouse was generated, its phenotype similar to that of persons with FRG [7]. SGLT1 is expressed predominantly in the intestine and to a lesser extent in the proximal tubule of the kidney [3], [4], [7]. Mutations in the *SLC5A1* gene encoding SGLT1 are found in persons with glucose-galactose malabsorption [3], [7]. Persons with this disorder have severe, life-threatening diarrhea due to hexose malabsorption [3], [7]. Attesting to the minor role for SGLT1 in renal glucose resorption under normoglycemic conditions, these persons exhibit only minimal glucosuria [8]. If treated with a glucose-galactose-deficient diet, the growth and development of these individuals can be normal [3].

Phlorizin, a nonselective inhibitor of SGLT1 and SGLT2, reduces BG in preclinical models of T2DM [9] and, due to its insulin-independent mechanism of action, also lowers BG in models of type 1 diabetes [9], [10]. Due to its nonselective nature and its poor pharmaceutical properties, phlorizin is unsuitable for clinical development [11]. Recently, a number of selective, metabolically stable SGLT2 inhibitors have been discovered and are in clinical development to treat T2DM [12]–[14].

Canagliflozin, one of these SGLT2 inhibitors, is currently in clinical development. In this report, we describe the selectivity and potency of canagliflozin and characterize its effects on UGE, RT_G_, glycemic control, glucose-stimulated insulin secretion, energy expenditure, and body weight in preclinical models of T2DM and insulin resistance.

## Materials and Methods

### Ethics Statement

All animal work was conducted according to relevant national and international guidelines. Full details of the study was approved by Johnson & Johnson PRD IASCUC committee (SH-MET-3011).

### Reagents and Chemicals

Canagliflozin was synthesized by Mitsubishi Tanabe Pharma Corporation, Medicinal Chemistry Laboratory (Toda, Saitama, Japan) [15]. Primers, vectors, and tissue culture media were purchased from Invitrogen (Carlsbad, California, USA). All other chemicals, unless otherwise specified, were purchased from Sigma (St. Louis, Missouri, USA).

### Cell-based Assays

Sodium-dependent Glucose Uptake in Chinese Hamster Ovary (CHO) Cells Expressing SGLT1 and SGLT2 Co-transporters Parental CHO-K (CHOK) cells (commonly used mammalian cells for gene overexpression studies) expressing human or mouse SGLT1 and SGLT2 were utilized in this study. Cells were seeded into 96-well plates. Cells were then washed one time with 0.15 ml assay buffer (137 mM NaCl, 5 mM KCl, 1 mM CaCl_2_, 1 mM MgCl_2_, 50 mM HEPES, pH 7.4) at 37°C. After the assay buffer was removed, 50 µl of fresh assay buffer with 5 µl of canagliflozin (0.3–300 nM) was added, followed by 10 minutes of incubation. Then, 5 µl of 6 mM alpha-methyl-d-glucopyranoside (AMG, a selective SGLT1/2 substrate)/^14^C-AMG (0.07 µCi) was added to the cells and incubated at 37°C for 2 hours. Next, the cells were washed 3 times with 0.15 ml ice-cold phosphate-buffered saline (PBS). After the final wash was aspirated, 50 µl of microscint 20 (Packard, Meriden, Connecticut, USA) was added. The plate was counted by TopCount (Packard, Meriden, Connecticut, USA).

#### The 2-deoxy-glucose (2-DG) Uptake in L6 Myoblast Cells

Cells from the rat skeletal muscle cell line, L6, was used to test the effect of canagliflozin on glucose transporter 1 (GLUT1) activity. Cells were maintained in Dulbecco's modified Eagle's medium containing 5.6 mM glucose supplemented with 10% fetal bovine serum, were seeded in 24-well plates at a density of 3.0×10^5^ cells/well and cultured for 24 hours in an atmosphere of 5% CO_2_ at 37°C. Cells were rinsed twice with Kreb's ringer phosphate HEPES buffer (pH 7.4, 150 mM NaCl, 5 mM KCl, 1.25 mM MgSO_4_, 1.25 mM CaCl_2_, 2.9 mM Na_2_HPO_4_, 10 mM HEPES) and were pre-incubated with the solutions of canagliflozin (250 µl, 10 uM) for 5 minutes at room temperature. The transport reaction was initiated by adding 50 µl of 4.5 mM 2-DG (a substrate for GLUTs)/^3^H-2-DG (0.625 µCi) followed by incubation for 15 minutes at room temperature. The 2-DG uptake was halted by aspiration of the incubation mixture. Cells were immediately washed 3 times with ice-cold PBS. Samples were extracted with 0.3 N NaOH, and radioactivity was determined by liquid scintillation (Packard, Boston, Massachusetts, USA).

#### Two-electrode Voltage Clamp Recording of Oocytes Expressing Human SGLT3

The functional effects of canagliflozin on human SGLT3 were studied by 2-electrode voltage clamp electrophysiology using OpusXpress 6000A (MDS Analytical Technologies, Ontario, Canada) [16]. Oocytes were obtained from Nasco (Fort Atkinson, Wisconsin, USA). Stage V–VI oocytes were injected with 50 nl of either human SGLT3 mRNA (at 1 ng/nl) or distilled water (control) and incubated at 18°C in a calcium-free solution (92 mM NaCl, 2 mM KCl, 1 mM MgCl_2_, 5 mM HEPES, 0.05 mg/ml gentamicin at pH 7.5) for 4–6 days before recording. The extracellular recording solution contained 92 mM NaCl, 2 mM KCl, 1.8 mM CaCl_2_, 1 mM MgCl_2_, and 5 mM HEPES at pH 7.5. Injected oocytes were impaled with 2 microelectrodes filled with 3 M KCl (resistance of ∼0.5–3 MΩ) and voltage clamped to −120 mV, at which continuous recordings were made (filtered at 5 kHz and sampled at 625 Hz). To establish the baseline in the absence of agonist, oocytes were first perfused for 85 seconds with a control buffer (92 mM NaCl, 2 mM KCl, 1.8 mM CaCl_2_, 1 mM MgCl_2_, 5 mM HEPES at pH 7.5). Next, 50 µM 1-deoxynojirimycin (DNJ) was applied for 160 seconds, followed by co-application of imino sugars 1-deoxynojirimycin (DNJ, 50 µM) with either canagliflozin (10 µM) or dimethyl sulfoxide (DMSO) (0.1%) for 160 seconds. Finally, phlorizin (3 mM) was applied in the presence of 50 µM DNJ for 160 seconds. All experiments were performed at 22°C. Currents in the presence of 50 µM DNJ were subtracted from leak currents (currents in control buffer alone) to obtain DNJ-induced currents (I_DNJ_). Effects of compounds were calculated as: % Inhibition = 100×(I_DNJ_−I_cmpd_)/I_DNJ_, where I_cmpd_ is the DNJ-induced and leak-subtracted current in the presence of a compound or DMSO. Due to lack of effect at the highest dose tested, a dose-response relationship was not examined.

### In Vivo Studies with Animal Models

#### Animals and Canagliflozin Administration

Four rodent models were used in these experiments: (1) male C57BL/6J mice fed with a high-fat diet (D-12492 with 60 kcal% fat, Research Diets, Inc, New Jersey, USA) (diet-induced obese, insulin resistantmice [DIO]); (2) male C57BL/ksj-db/db hyperglycemic mice; (3) male Zucker fatty (ZF) obese, insulin resistant rats; and (4) male ZDF obese, hyperglycemic rats. All animals were housed according to standard procedures following a protocol approved by the IACUC in Drug Discovery at Johnson & Johnson Pharmaceutical Research & Development, LLC. Canagliflozin was formulated in 0.5% hydroxypropyl methylcellulose and administrated via oral gavage at 10 ml/kg.

#### Renal Glucose Handling

Two separate graded glucose infusion (GGI) studies were performed to characterize renal glucose handling in both untreated and treated ZDF rats. In both experiments, rats were anesthetized with thiobutabarbital (100 mg/kg); a catheter was inserted into the jugular vein for glucose infusion; another catheter was introduced into the bladder to collect urine; and a graded GGI was performed (infusion rate increased from 1.5 ml/kg/h to 6.0 ml/kg/h of 10% glucose over 90 min) to increase BG levels. In the first study, the GGI increased BG from overnight-fasted values of approximately 200 mg/dl to approximately 475 mg/dl. In the second experiment, insulin was infused (3 U/ml, 0.7 ml/kg/h) for 60 minutes to lower BG levels to approximately 25 mg/dl, and then the GGI was performed to increase BG to approximately 300 mg/dl over 90 minutes. Blood and urine samples were collected for each 5-min interval to measure glucose and creatinine levels. The RT_G_ was determined as described below.

#### Determining RT_G_

The relationship between UGE and BG is commonly described as a threshold relationship in which there is little UGE when BG<RT_G_ and UGE increases with filtered glucose load when BG>RT_G_, where the filtered glucose load is equal to the BG concentration multiplied by the glomerular filtration rate (GFR). The idealized threshold relationship in which UGE is 0 whenever BG is below RT_G_, and then increases with the filtered glucose load when BG>RT_G_, can be written mathematically as [17], [18]:
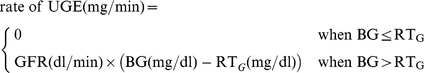
(1)Values of RT_G_ were determined using nonlinear regression (nlinfit in Matlab version 7.10) with Equation 1 and the measured BG and UGE values.

#### Reduction of Hyperglycemia in Diabetic Rodent Models

To examine the effect of canagliflozin on hyperglycemia, single doses of canagliflozin (0.1, 1, and 10 mg/kg) were administered to overnight-fasted db/db mice. BG levels were monitored at 0, 0.5, 1, 3, 6, and 24 hours after dosing. Canagliflozin was also administered to ZDF rats at varying doses (3–30 mg/kg) for 4 weeks to evaluate its effect on BG control and pancreatic beta-cell function. BG levels were monitored weekly, and HbA1c, plasma glucose, and insulin levels were determined at the end of the 4-week treatment. An oral glucose tolerance test (OGTT) (2 mg/kg of body weight, given by gavage) was conducted in ZDF rats after 4 weeks of treatment. Blood was sampled at 0, 30, 60, and 120 minutes after glucose challenge from the tail vein for measurement of BG levels using a glucometer (One Touch Ultra, LifeScan, Milpitas, California, USA) and plasma insulin using ELISA method (ALPCO, Salem, New Hampshire, USA).

#### Body Weight Control Studies in Obese Mice and Rats

The effects of canagliflozin on body weight gain were evaluated in DIO mice and ZF rats. DIO mice received a 4-week treatment of canagliflozin at 30 mg/kg. Body weight, food intake, and BG levels were monitored weekly. UGE and indirect calorimetry were conducted in the fourth week of treatment during the compound treatment. In another study, ZF rats were treated with canagliflozin at 3 mg/kg for 3 weeks. Body weight, food intake, and BG were measured weekly during the 19-day treatment period. UGE, body fat, and indirect calorimetric studies were conducted at the end of this study.

#### Data Analysis

Statistical analysis was performed using the program Prism (Graphpad, Monrovia, California, USA). Differences between groups were evaluated using 1-way analysis of covariance with Dunnett's multiple comparison test or student's *t*-test (unpaired), as appropriate. Probabilities less than 5% (P<0.05) were considered to be statistically significant. Fifty-percent inhibitory concentration (IC_50_) and 95% confidence interval (CI) were determined by nonlinear least-squares analysis using a 4-parameter logistic model. All data are presented as mean ± standard error.

## Results

### Effect of Canagliflozin on AMG Uptake in CHO Cells Expressing SGLTs and 2-DG Uptake in L6 Myoblast Cells

**Figure 1:**
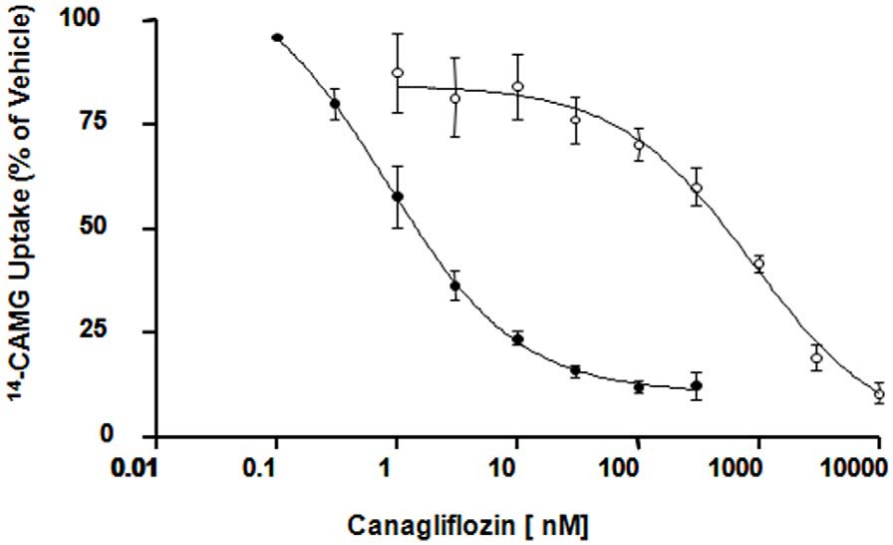
Inhibitory effects of canagliflozin on human SGLT1 and human SGLT2. The inhibitory effect of canagliflozin on ^14^C-AMG uptake in CHOK-hSGLT1 and CHOK-hSGLT2 has been measured in 5 experiments. A typical inhibitory effect on CHOK-hSGLT1 (Panel A) and CHOK-hSGLT2 (Panel B) from a single experiment is presented here.

In a concentration-dependent fashion, canagliflozin inhibited Na^+^-dependent ^14^C-AMG uptake in CHO-hSGLT2 cells, with an IC_50_ of 4.4±1.2 nM. Similar IC_50_ values were obtained in CHO-rSGLT2 and CHO-mSGLT2 cells (IC_50_ = 3.7 and 2.0 nM for rat and mouse SGLT2, respectively). Canagliflozin inhibited ^14^C-AMG uptake in CHO-hSGLT1 and mSGLT1 cells with IC_50_ of 684±159 nM and >1,000 nM, respectively. The IC_50_ values for hSGLT1 and hSGLT2 are similar to previously reported values for canagliflozin [19]. Figure 1 represents results from a typical experiment. At 10 µM, canagliflozin inhibited the facilitative (non-Na^+^-linked) GLUT-mediated 3H-2-DG uptake in L6 myoblasts by less than 50% (Table 1).

**Table 1:** Effects of canagliflozin on SGLT1-, SGLT2-, and facilitative glucose transporter-mediated glucose transport, and on SGLT3-induced currents.

Cells line	SGLT tested	Substrate for transporter	IC_50_, nM
CHOK cells	Human SGLT1	^14^C-AMG	684±159
	Human SGLT2	^14^C-AMG	4.4±1.2
	Rat SGLT1	^14^C-AMG	571
	Rat SGLT2	^14^C-AMG	3.7
	Mouse SGLT1	^14^C-AMG	>1000
	Mouse SGLT2	^14^C-AMG	2.0
Rat L6 myoblast cells		^3^H-2-DG	>10,000
Oocytes	Human SGLT3	DNJ-induced currents	No inhibitory effect

CHOK cells over-expressed with human, rat, or mouse SGLT1 or SGLT2 and rat L6 myoblast cells were used. AMG or 2-DG uptake was determined and IC_50_ values were calculated as described in Methods section. Data of human SGLT1 and SGLT2 were presented the summary of 5 studies. Other data presented the mean value of 2 experiments. Oocytes expressed with human SGLT3 were used to determine canagliflozin effect on DNJ-induced current.

2-DG, 2-deoxy-d-glucose; AMG, alpha-methylglucoside; CHOK, Chinese hamster ovary-K; IC_50_, concentration required to inhibit 50% of activity; SGLT, sodium glucose co-transporter; DNJ, imino sugars 1-deoxynojirimycin.

### Effects of Canagliflozin on hSGLT3 Exogenously Expressed in Oocytes

It has been reported recently that SGLT3 functions as a glucose sensor [20]. To explore the effects of canagliflozin on the function of this protein, human SGLT3 was expressed in oocytes. In sham (water)-injected oocytes, imino sugars 1-deoxynojirimycin (DNJ, 50 µM) did not induce currents. By contrast, ooctyes injected with human SGLT3 mRNA expressed significant DNJ-sensitive currents (−1.6±1.1 nA [n = 14] for sham-injected versus 43.4±2.9 nA [n = 60] for SGLT3-injected oocytes; P<0.05). In sham-injected oocytes, canagliflozin (10 µM) or phlorizin (3 mM) alone in the presence of 50 µM DNJ did not affect currents. In SGLT3-injected oocytes, DMSO and canagliflozin 10 µM inhibited DNJ-induced currents by 15.6±3.1% (n = 5) and 23.4±4.3% (n = 9), respectively. The difference between DMSO and canagliflozin treatments was not statistically significant. In contrast, phlorizin (3 mM) caused significant (P<0.05) inhibition of DNJ-induced currents in SGLT3-injected oocytes (73.5±4.2%; n = 14; Table 1).

### Effect of Canagliflozin on the Relationship between BG and UGE

**Figure 2:**
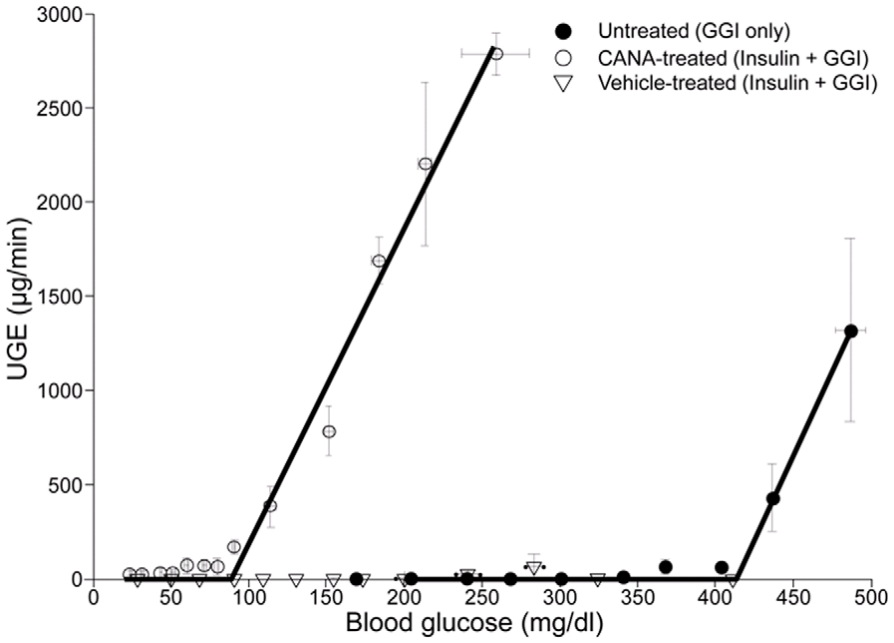
BG and UGE in untreated and CANA-treated ZDF rats during two GGI studies. In the first study (GGI only), a GGI was given to untreated ZDF rats (*n* = 8) to raise BG from 212±24 mg/dl to ∼475 mg/dl over 90 minutes. In the second study (Insulin+GGI), ZDF rats (*n* = 6/group) were treated with either vehicle or canagliflozin (1 mg/kg), then given insulin to lower BG to 25±1 mg/dl, and finally given the GGI to slowly raise BG. BG and UGE were measured every 5 minutes. Results shown are mean ± S.E. GGI, graded glucose infusion; BG, blood glucose; UGE, urinary glucose excretion; ZDF, Zucker diabetic fatty rats; GG1, graded glucose infusion.

Two separate GGI studies were performed to characterize the relationship between BG and UGE in untreated and treated ZDF rats. In the first study, untreated ZDF rats given a GGI to raise BG from approximately 200 mg/dl to approximately 475 mg/dl did not experience appreciable UGE until BG levels were >400 mg/dl and RT_G_ = 415±12 mg/dl. Canagliflozin treatment (1 mg/kg) notably lowered RT_G_ in ZDF rats to 94±10 mg/dl (Figure 2). In the second study, an insulin infusion was given to lower BG to approximately 25 mg/dl, and then the GGI was given to slowly raise BG to approximately 300 mg/dl. In ZDF rats treated with canagliflozin (1 mg/kg), the relationship between BG and UGE remained well-described by a threshold relationship with negligible UGE occurring when BG<90 mg/dl and UGE increased in proportion to BG at higher BG levels, whereas virtually no UGE was observed in vehicle-treated animals in this study (Figure 2).

### Effect of Canagliflozin on Glycemic Control in db/db Mice and ZDF Rats

In db/db mice, single doses of canagliflozin dose-dependently reduced non-fasting BG concentrations. The onset of the BG-lowering effect after a single dose was rapid, and BG levels in canagliflozin-treated mice (at 1 and 10 mg/kg doses) were significantly different from those of vehicle-treated mice at 1 hour after treatment (22% and 36% reduction of BG levels compared with that in vehicle-treated mice [P<0.5], respectively). This antihyperglycemic effect reached its peak at 6 hours (BG levels at 208±9.6 or 118±9.0 mg/dl in db/db mice treated with 1 or 10 mg/kg, respectively, compared with BG level of 479±3.9 mg/dl in vehicle-treated mice). To evaluate the chronic effect of canagliflozin on glycemic control, ZDF rats were treated with the compound at doses of 3, 10, or 30 mg/kg for 4 weeks. The non-fasting BG and HbA1c levels decreased in all canagliflozin-treated rats compared with the vehicle-treated group (Table 2). Canagliflozin treatment also reduced BG levels in ZDF rats following an OGTT (Table 2) after 4 weeks of treatment. Canagliflozin treatment improved beta-cell function as demonstrated by a 4-to-6-fold increase, relative to vehicle-treated animals, in the insulin/glucose ratio following an OGTT (Table 2).

**Table 2:** Effects of a 4-week treatment of canagliflozin on blood glucose and insulin levels in ZDF rats.

	Vehicle	3 mg/kg	10 mg/kg	30 mg/kg
Fed plasma glucose (mg/dl)	598.2±18.0	248.0±13.5^a^	182.0±7.1^a^	199.9±14.7^a^
HbA1c (%)	11.5±0.3	7.1±0.3^a^	5.6±0.3^a^	5.5±0.3^a^
Insulin (ng/ml)	5.2±0.4	7.9±0.1^a^	7.9±0.1^a^	7.8±0.1^a^
OGTT				
Blood glucose (AUC_0–120 min_, mg/dl•min)	70,704±1289	27,883±1806^a^	23,989±1881^a^	16,549±822^a^
Plasma insulin (AUC_0–120 min_, ng/ml•min)	221±13	342±27^a^	403±33^a^	288±34
Ratio of insulin AUC/glucose AUC (ng/ml•min)/mg/dl•min)	0.003±0.000	0.013±0.002^a^	0.017±0.002^a^	0.018±0.002^a^

ZDF rats were treated with different doses of canagliflozin or vehicle for 4 weeks. Fed plasma glucose levels, HbA1c, and plasma insulin were determined at the end of this study. In addition, an OGTT was conducted. Blood glucose, plasma insulin, and urine glucose excretion were measured during OGTT. Data are presented as mean ± SEM (*n* = 8).

AUC, area under the curve; HbA1c, glycated hemoglobin; OGTT, oral glucose tolerance test; ZDF, Zucker diabetic fatty.

a *p*<0.05 compared with vehicle-treated group.

### Effect of Canagliflozin on Body Weight Gain in DIO Mice and ZF Rats

In DIO mice, canagliflozin 30 mg/kg treatment for 4 weeks reduced BG levels, respiratory exchange ratio, and body weight gain (Table 3). In ZF rats treated with canagliflozin 3 mg/kg for 3 weeks, UGE was increased with no significant change in total food intake compared with that in vehicle-treated rats, leading to a decrease in body weight. Relative to vehicle-treated ZF rats, canagliflozin-treated ZF rats had a reduction in respiratory exchange ratio. The weight of epididymal fat pads and liver tissues in compound-treated groups tended to be reduced. Feeding efficiency, defined as the body weight gain divided by the food intake, decreased in canagliflozin-treated ZF rats (Table 3).

**Table 3:** Effects of canagliflozin on body weight, glycemia, UGE, and respiratory exchange ratio in DIO mice and ZF rats.^a^

Treatment	ZF rat		DIO mouse	
	Vehicle	3 mg/kg	Vehicle	30 mg/kg
Body weight (g)	437.9±11.5	408.1±7.7^b^	53.2±0.6	49.6±1.0^b^
Total food intake (g)	645.3±21.2	647.9±18.9	ND	ND
Feeding efficiency index (body weight gain/food intake)	0.30±0.01	0.25±0.01^†^	ND	ND
Fed BG (mg/dl)	149.6±22.9	118.9±4.3^b^	199±16	105±13^b^
UGE (mg/4 h)	1.8±1.1	29.10±3.5^b^	2.4±0.6	26.1±5.2^b^
RER	1.10±0.01	1.06±0.01^b^	0.79±0.01	0.77±0.01^b^
Oxygen consumption (VO_2_, ml/kg•h)	1560±28	1582±33	2364±29	2419±109
Epididymal fat weight (g)	10.8±0.3	9.5±0.3^b^	ND	ND
Liver weight (g)	28.5±0.8	25.0±1.4^b^	ND	ND

DIO mice were treated with either vehicle or canagliflozin 30 mg/kg for 4 weeks. Body weight was monitored twice per week. BG, UGE, and energy expenditure were measured at the end of this study.

BG, blood glucose; DIO, diet-induced obesity; ND, not detected; RER, respiratory exchange ratio; UGE, urinary glucose excretion; VO_2_, oxygen consumption; ZF, Zucker fatty.

a ZF rats were treated with either vehicle or canagliflozin 3 mg/kg for 3 weeks. Body weight and food intake were monitored twice per week. BG, UGE, and energy expenditure were measured at the end of this study. In addition, the weight of epididymal fat pad and liver tissue were determined during necropsy. Data are presented as mean ± SEM (*n* = 8).

b *p*<0.05 compared with vehicle-treated group.

## Discussion

Based on in vitro assessments, canagliflozin is a potent and selective SGLT2 inhibitor. By inhibiting SGLT2, canagliflozin lowers the renal glucose resorptive capacity and increases UGE. The GGI studies performed demonstrate that the relationship between BG and UGE in canagliflozin-treated animals remains well described by a threshold relationship such that UGE in canagliflozin-treated animals remained near zero when BG levels were below RT_G_, and increased as BG increased when BG levels were above RT_G_. Canagliflozin treatment (1 mg/kg) lowered RT_G_ in ZDF rats from 415±12 mg/dl to 94±10 mg/dl. RT_G_ values in the untreated ZDF rats were considerably higher than values of 180–200 mg/dl commonly reported in humans [21], [22]. The higher RT_G_ values observed in the ZDF rats compared with humans may be due in part to interspecies differences but may also be due to increased expression of renal glucose transporters that has been reported in diabetic rodents [23]–[25] and humans [26]. Consistent with the elevated renal glucose transporter expression, increased renal glucose re-absorptive capacity has been reported in humans with type 1 [27] and type 2 [28] diabetes.

In preclinical models of insulin resistance and diabetes, SGLT2 inhibitors improve beta-cell function and preserve islet morphology [29], [30]. As assessed using insulin/glucose ratio during the OGTT, canagliflozin improved beta-cell function in ZDF rats. SGLT2 inhibition may indirectly improve beta-cell function by reducing the toxic effects of hyperglycemia on the beta cell (i.e., reducing glucotoxicity) [31]. By reducing hyperglycemia via a non–insulin-dependent mechanism, SGLT2 inhibition decreases the demand on beta cells to secrete insulin. Increased insulin secretory demand results in endoplasmic reticulum stress, which in turn can lead to increases in beta-cell apoptosis [32]. A reduction in insulin secretory demand and, consequently, endoplasmic reticulum stress, could reduce beta-cell apoptosis, a condition increased in patients with T2DM [33]. Thus, by reducing demand on beta cells to secrete insulin as well as reducing glucotoxicity, SGLT2 inhibitors theoretically could reduce the rate of progression of T2DM [34]–[37].

In DIO mice and ZF insulin-resistant rats, caloric loss due to canagliflozin-induced increases in UGE was associated with reductions in body weight gain, epididymal fat mass, and hepatic weight. Additionally, a reduction in the respiratory exchange ratio, suggestive of increased fatty acid metabolism and/or reduced de novo lipogenesis, was observed in canagliflozin-treated animals. No compensatory increase in food consumption was detected in animals treated with canagliflozin. Although not directly assessed in this study, SGLT2 inhibitor-induced weight loss could indirectly improve insulin sensitivity. The reduction in liver weight and in respiratory quotient (RQ) observed in our studies are consistent with observations in gene-targeted mice lacking SGLT2 that indicate in mice lacking SGLT2 and fed with high-fat diet an increase in lipid oxidation, a reduction in hepatic glycogen content, and a decrease in RQ [38] relative to wild-type mice. In preclinical models of insulin resistance, improvements in insulin sensitivity, especially in hepatic insulin sensitivity, have been noted in animals treated with SGLT2 inhibitors [39].

A number of SGLT2 inhibitors, including canagliflozin, are in late-stage clinical development [12]–[14]. Many of the preclinical pharmacology findings with canagliflozin have been recapitulated in clinical trials with canagliflozin and other selective SGLT2 inhibitors. For instance, in patients with T2DM, treatment with SGLT2 inhibitors has led to improved glycemic control, reductions in body weight, and improvements in beta-cell function [40]–[43].

In summary, our current studies demonstrated that canagliflozin is a potent and selective SGLT2 inhibitor that lowers RT_G_, increases UGE, improves glycemic control, improves beta-cell function, reduces body weight, increases fatty acid oxidation, and reduces de novo lipogenesisin rodent models of insulin resistance and T2DM. These preclinical data suggest that canagliflozin may provide benefits beyond improving glycemic control in the treatment of T2DM and support the clinical development of canagliflozin to more fully characterize the benefit-risk profile of this agent in treating T2DM.
